# Uracil DNA glycosylase interacts with the p32 subunit of the replication protein A complex to modulate HIV-1 reverse transcription for optimal virus dissemination

**DOI:** 10.1186/s12977-016-0257-x

**Published:** 2016-04-12

**Authors:** Cecile Herate, Clarisse Vigne, Carolin A. Guenzel, Marie Lambele, Marie-Christine Rouyez, Serge Benichou

**Affiliations:** Inserm U1016, Institut Cochin, 22 Rue Méchain, 75014 Paris, France; CNRS, UMR8104, Paris, France; Université Paris-Descartes, Sorbonne Paris-Cité, Paris, France

**Keywords:** HIV-1, Vpr, UNG2, RPA32, Reverse transcription

## Abstract

**Background:**

Through incorporation into virus particles, the HIV-1 Vpr protein participates in the early steps of the virus life cycle by influencing the reverse transcription process. We previously showed that this positive impact on reverse transcription was related to Vpr binding to the uracil DNA glycosylase 2 enzyme (UNG2), leading to enhancement of virus infectivity in established CD4-positive cell lines via a nonenzymatic mechanism.

**Results:**

We report here that Vpr can form a trimolecular complex with UNG2 and the p32 subunit (RPA32) of the replication protein A (RPA) complex and we explore how these cellular proteins can influence virus replication and dissemination in the primary target cells of HIV-1, which express low levels of both proteins. Virus infectivity and replication in peripheral blood mononuclear cells and monocyte-derived macrophages (MDMs), as well as the efficiency of the viral DNA synthesis, were significantly reduced when viruses were produced from cells depleted of endogenous UNG2 or RPA32. Moreover, viruses produced in macrophages failed to replicate efficiently in UNG2- and RPA32-depleted T lymphocytes. Reciprocally, viruses produced in UNG2-depleted T cells did not replicate efficiently in MDMs confirming the positive role of UNG2 for virus dissemination.

**Conclusions:**

Our data show the positive effect of UNG2 and RPA32 on the reverse transcription process leading to optimal virus replication and dissemination between the primary target cells of HIV-1.

## Background

Human immunodeficiency virus type 1 (HIV-1) has two main target cells, T CD4-positive lymphocytes and myeloid cells, including macrophages and dendritic cells. Infection of the CD4-positive T lymphocytes leads to the depletion of this cell population during the acute phase of the disease while macrophages are identified as one of the major reservoirs of virus [1]. HIV-1-infected macrophages actively participate in virus dissemination and in the establishment of persistent virus reservoirs in different host tissues including lungs, gastro-intestinal and genital tracts, and the central nervous system (CNS) [2, 3]. Strains of HIV-1 have the ability to enter and infect different cell types in vitro and are usually subdivided into three main groups according to their co-receptor usage. Some viral strains use the beta-chemokine receptor CCR5 (R5 strains) and can infect monocytes and macrophages but also PBMCs and primary T lymphocytes. Other viral strains, emerging later during the infection, preferentially enter T lymphocytes and established T cell lines via the alpha-chemokine receptor CXCR4 (X4 strains) and are associated with progression to AIDS (for review, [4]). Dual tropic strains with both co-receptor usages also exist and can thus infect all the conventional HIV-1 target cells.

Like other viruses, HIV-1 utilizes or perturbs cellular pathways in order to optimize essential steps of the virus life cycle in T lymphocytes, macrophages or dendritic cells. Moreover, the HIV-1 genome contains additional genes encoding regulatory proteins Nef, Vif, Vpu and Vpr, which are viral factors specialized in hijacking and perturbing essential cellular pathways during virus replication through interactions with host cell proteins. However, Vpr is the only HIV-1 auxiliary protein specifically incorporated into virus particles through direct interaction with the Pr55Gag precursor, since its presence in the virion core will be subsequently required during the early steps of the virus life cycle in the newly infected cell (for review, [5]).

After virus entry, the viral core is released into the cytoplasm of the target cell where reverse transcriptase synthesizes the viral DNA, and the first role of Vpr is to influence the accuracy of the reverse transcription process leading to modulation of the mutation rate in the newly synthesized viral DNA. Vpr may also contribute to the mechanisms that allow the viral DNA to access the nuclear compartment. In addition, Vpr is a multifunctional protein that displays other activities including perturbations of the cell cycle progression, induction of apoptosis, and transcriptional modulation of host cell genes [5].

We and others have previously shown that the role of Vpr in the modulation of the reverse transcription process is related to the direct interaction and subsequent recruitment into virus particles of the nuclear form of uracil DNA glycosylase (UNG2) [6–9]. However, the specific role of UNG2 incorporation into virions was also challenged by other studies [10–12], suggesting that UNG2 had either a detrimental [11, 12] or a dispensable role in virus replication [10]. UNG2 is a base excision repair enzyme mainly involved in removal of uracil residues from DNA at the replication fork during chromosome replication [13]. The inclusion of uracil residues in DNA can occur either by mis-incorporation of dUTP during replication or by cytosine deamination. UNG2 is able to bind other key proteins of the DNA repair machinery such as the p32 subunit (RPA32) of the replication protein A complex (RPA), the proliferating cell nuclear antigen (PCNA) and the X-ray repair cross-complementing group 1 (XRCC1) [14–20]. The RPA complex is able to bind single-strand DNA (ssDNA) and can protect DNA intermediates during DNA replication and even stabilize DNA fragments caused by DNA damage [21, 22]. Interestingly, it was reported that the RPA complex promotes the recruitment of UNG2 at the replication fork [21, 22].

UNG2 has been also largely studied for its critical role in somatic hypermutation (SHM) and class-switch recombination (CSR) at the immunoglobulin locus of B lymphocytes [13]. While controversial (for review, Refs. [13, 23, 24]), some reports showed that catalytically inactive mutants of UNG2 were fully proficient in CSR in B lymphocytes [25–27] suggesting that the specific function of UNG2 in CSR was not related to the catalytic activity of the protein but depends on a novel nonenzymatic function of UNG2. Similarly, we have recently reported that the catalytic activity of UNG2 is not required for modulation of the HIV-1 reverse transcription process and its positive impact on virus infectivity. This function of UNG2 is related to determinants located within the N-terminal region of the protein that is required for direct interaction with the RPA32 subunit of the RPA complex [7]. Interestingly, it was recently reported that the RPA complex also played a specific role during CSR [28].

Here, we report results showing the positive impact of UNG2 and RPA32 on the reverse transcription leading to optimal virus infectivity, replication and dissemination between HIV-1 primary target cells. Interestingly, primary cells such as peripheral blood mononuclear cells (PBMCs) and monocyte-derived macrophages express low levels of these two cellular proteins.

## Results

### Vpr, UNG2 and RPA32 can be associated together in a trimolecular complex

**Fig. 1:**
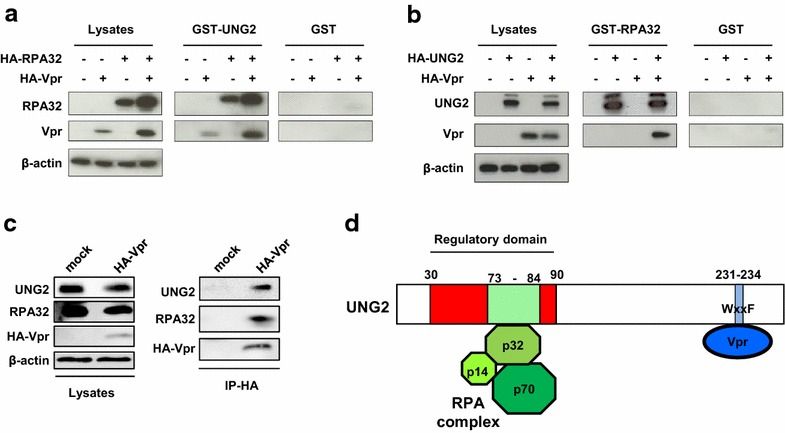
Characterization of the Vpr/UNG2/RPA32 molecular complex. **a**, **b** In vitro binding analyses of Vpr/UNG2/RPA32 interactions. 293T cells were cotransfected with plasmids for expression of HA-tagged forms of Vpr, UNG2 and RPA32. Lysates from transfected cells were then incubated with 5 µg of GST, GST-UNG2 (**a**) or GST-RPA32 (**b**) immobilized on GSH-Sepharose beads. Bound proteins were resolved by SDS-PAGE and analyzed by Western blot with anti-HA and anti-β-actin antibodies. Equal amount of cell lysate proteins from transfected cells was run as control on the *left panels*. **c** Co-immunoprecipitation of the Vpr/UNG2/RPA32 complex. 293T cells were tranfected with the HA-Vpr expression plasmid or the control plasmid (mock). Cells were lyzed 48 h later and Vpr was precipitated with anti-HA antibody. Immunoprecipitates (*right panels*) and cell lysates (*left panels*) were then analyzed by Western blotting with anti-HA, anti-UNG2, anti-RPA32 and anti-β-actin antibodies. **d** Schematic representation of UNG2 showing the interaction domains with Vpr and the RPA32 (p32) subunit of the RPA complex. The 231–234 WxxF motif of UNG2 (indicated in *blue*) interacts with Vpr [35] while the N-terminal part of UNG2 encompassing amino-acids 73–84 (in *green*) contains determinants for RPA32 binding [16, 18, 19]

Previously, we reported that the ability of UNG2 to modulate the HIV-1 mutation rate was dependent on a 60-amino-acid domain located in the N-terminal regulatory region (amino acids 30–90) of the cellular protein containing the determinants for direct interaction with the RPA32 (i.e. p32) subunit of the RPA complex [7] (see on Fig. 1d). Therefore, RPA32 expression was shown to increase virus infectivity in a single-round infectivity assay. In order to further document the role of these two cellular proteins in HIV-1 replication, we first evaluated whether they could assemble to form a complex with the HIV-1 Vpr protein. We used in vitro binding assays performed with recombinant UNG2 and RPA32 expressed in *E. coli* in fusion with the glutathione S-transferase (GST-UNG2 and GST-RPA32, Fig. 1a, b, respectively). Purified recombinant GST-UNG2 and GST-RPA32 were immobilized on glutathione (GSH)-Sepharose beads and then incubated with lysates from 293T cells expressing hemagglutinin (HA)-tagged forms of Vpr, UNG2 and RPA32, either alone or in combination. Bound proteins were then analyzed by Western blotting with anti-HA. As expected, both HA-Vpr and HA-RPA32 specifically bound to GST-UNG2 but not to GST, when they are expressed alone or in combination (Fig. 1a). Similarly, both HA-Vpr and HA-UNG2 were able to bind to GST-RPA32 when they were expressed in combination (Fig. 1b). However, HA-Vpr expressed alone did not bind to GST-RPA32 (Fig. 1b), indicating that UNG2 acts as a linker between RPA32 and Vpr to form a trimolecular complex containing Vpr, UNG2 and RPA32, as schematized on Fig. 1d. Finally, we demonstrated that endogenous UNG2 and RPA32 proteins could associate together with HA-Vpr by a co-immunoprecipitation assay. HA-Vpr expressing cells were lysed and Vpr was immunoprecipitated with an anti-HA antibody. As shown in Fig. 1c, endogenous UNG2 and RPA32 were detected only in the precipitate from lysate of cells expressing HA-Vpr but not from mock cell lysate.

### UNG2 and RPA32 are required for efficient HIV-1 replication in established human cell lines

**Fig. 2:**
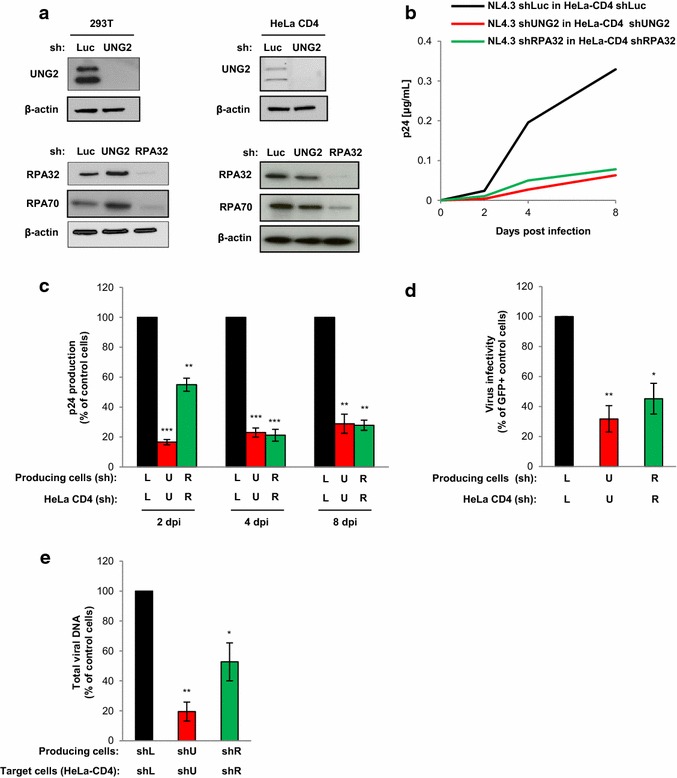
Impact of UNG2 and RPA32 depletion on HIV-1 replication in HeLa-CD4 cells. **a** Depletion of UNG2 and RPA32 in 293T (*left panels*) and HeLa-CD4 (*right panels*) cells. Cells were transduced with lentiviral vectors expressing shRNA against UNG2, RPA32 or Luciferase (Luc) used as a control. Lysates from shRNA-transduced cells were analyzed by Western blot using anti-UNG2, anti-RPA32, anti-RPA70 and anti-β-actin antibodies. **b**, **c** Virus replication in UNG2- or RPA32-depleted cells. Replication-competent viruses were produced in UNG2-, RPA32-depleted or in control shLuc 293T cells, normalized for viral p24, and then used for infection of UNG2-depleted (*red line* and *bars*), RPA32-depleted (*green line* and *bars*) or control shLuc (*black line* and *bars*) HeLa-CD4 cells. Aliquots of cell culture supernatant were collected 2, 4, and 8 days after infection for p24 quantification. In **b**, the kinetic of replication shown is representative of four independent experiments. In **c**, results are the means of the four independent experiments and are expressed as the percentage of p24 production at each time point relative to that of shLuc-transduced HeLa-CD4 cells infected with control viruses. **d** Virus infectivity. Wild-type GFP reporter viruses were produced in shUNG2-, shRPA32- or shLuc-transduced 293T cells, normalized for p24, and then used to infect shUNG2-, shRPA32- or shLuc-transduced HeLa-CD4 cells as indicated. The percentage of GFP-positive infected cells was then measured by flow cytometry 60 h later. Viral infectivity was normalized to that of viruses produced in control 293T cells and measured on control HeLa-CD4 as target cells. **e** Quantification of total viral DNA. Infected HeLa-CD4 cells were collected 7 h after infection, subjected to DNA purification, and the total viral DNA was quantified by qPCR using specific primers for *U5*-*gag*. Results are expressed as the percentage of total viral DNA relative to that of shLuc-transduced HeLa-CD4 cells infected with control viruses produced in shLuc-transduced 293T cells. Values are the means of at least three independent experiments. *Error bars* represent 1 SEM (standard error of the mean). Statistical significance was determined using Students *t* test (ns, p > 0.05; *p < 0.05; **p < 0.01; ***p < 0.001)

First, we analyzed how UNG2 and RPA32 might affect virus replication in human established cell-lines. Replication-competent viruses were produced in 293T cells depleted of UNG2 or RPA32 with specific shRNAs, and used to infect target HeLa-CD4 cells also depleted for UNG2 or RPA32 with the same shRNAs. As evidenced by Western blot analysis of cell lysates from shRNA-transduced 293T and HeLa-CD4 cells (Fig. 2a, left and right panels, respectively), the UNG2 protein bands of 37–39 kDa as well as the RPA32 protein band of 32 kDa were significantly reduced in lysates from shUNG2- or shRPA32-transduced cells but not from shLuc-transduced control cells. Moreover, we also noticed an important decrease of the 70 kDa subunit (RPA70) expression in shRPA32-transduced cells suggesting a destabilization of the whole RPA complex through depletion of the RPA32 subunit (Fig. 2a, lower panels).

As shown in Fig. 2b, c, the depletion of UNG2 in HeLa-CD4 cells led to a drastic decrease of virus replication as measured by the concentration of the viral p24 capsid protein (p24) in the cell-culture supernatant. This impairment in virus replication in shUNG2-transduced HeLa-CD4 cells (red curve and red bars, respectively) was observed as soon as 2 days post-infection and remained significant 4 and 8 days post-infection compared to shLuc-transduced HeLa-CD4 control cells (black curve and black bars). The requirement of the RPA32 protein for HIV-1 replication in HeLa-CD4 cells was similarly analyzed (Fig. 2b, c). Compared to control viruses produced in shLuc-transduced 293T cells and used to infect shLuc-transduced control HeLa-CD4 cells (black curve and black bars), viruses produced in RPA32-depleted cells also failed to replicate efficiently in RPA32-depleted HeLa-CD4 target cells (green curve and green bars). Together, these results clearly show the requirement of UNG2 and RPA32 proteins in both producing and target cells to ensure efficient virus replication. Furthermore, as previously reported [7], a significant decrease in virus infectivity, evaluated in a single-round infection assay with non-replicative GFP reporter viruses, was observed when viruses were produced in UNG2- and RPA32-depleted HeLa-CD4 cells (Fig. 2d), suggesting that incorporation of UNG2 and RPA32 into viral particles is required for maintaining full HIV-1 infectivity in this single-round infection assay. In order to confirm that the defect in virus replication in UNG2- and RPA32-depleted cells was related to a defect in the reverse transcription (RT) process, total viral DNA reverse transcripts were quantified 7 h after infection of HeLa-CD4 cells. As shown in Fig. 2e, a significant reduction in viral DNA synthesis was observed in UNG2- (red bar) and RPA32-depleted (green bar) cells compared to shLuc-transduced control cells (black bar).

**Fig. 3:**
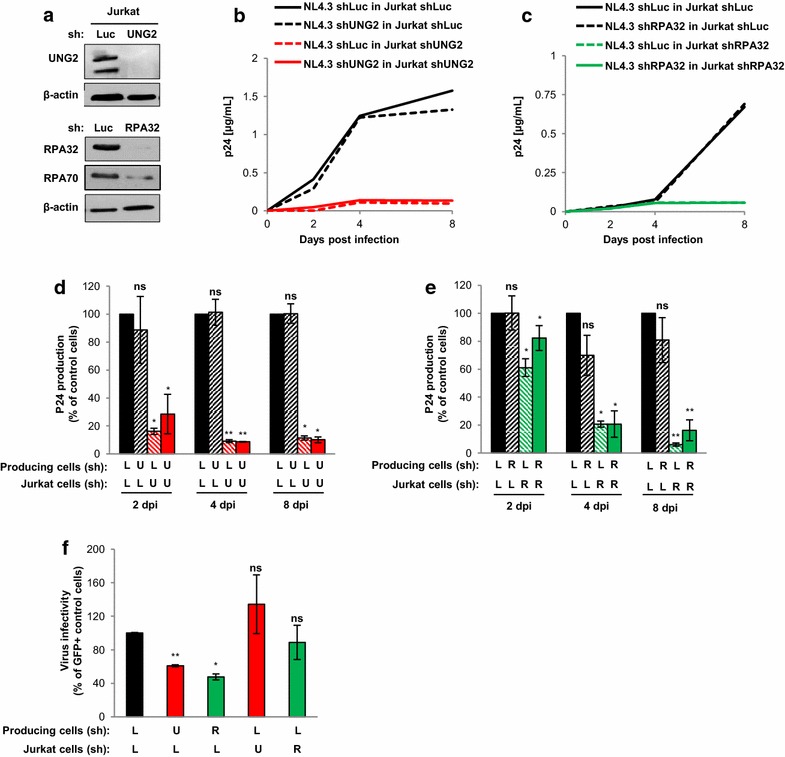
Impact of UNG2 and RPA32 on HIV-1 replication in Jurkat T cells. **a** Depletion of UNG2 and RPA32 in Jurkat cells. Cells were transduced with lentiviral vectors expressing shRNA against UNG2, RPA32 or Luciferase. Lysates from shRNA-transduced cells were analyzed by Western blot using anti-UNG2, anti-RPA32, anti-RPA70 and anti-β-actin antibodies. **b**–**e** Virus replication in UNG2- or RPA32-depleted Jurkat cells. Replication-competent viruses were produced in UNG2- (B and D) or RPA32- (**c**, **e**) depleted cells or in control shLuc-transduced 293T cells, normalized for p24, and then used for infection of shLuc-, shUNG2- or shRPA32-transduced Jurkat cells. Aliquots of cell culture supernatant were collected 2, 4, and 8 days after infection for p24 quantification. In **b**, **c**, the kinetic of replication shown is representative of four independent experiments. In **d**, **e**, values are the means of the four independent experiments. Results are expressed as the percentage of p24 production at each time point relative to that of shLuc-transduced Jurkat cells infected with viruses produced in shLuc-transduced 293T cells. **f** Virus infectivity in UNG2- and RPA32-depleted cells. Wild-type GFP reporter viruses were produced in shUNG2-, shRPA32- or shLuc-transduced 293T cells, normalized for p24, and then used to infect shUNG2-, shRPA32- or shLuc-transduced Jurkat cells as indicated. The percentage of GFP-positive infected cells was then measured by flow cytometry 60 h later. Viral infectivity was normalized to that of viruses produced in shLuc-transduced 293T cells and measured on shLuc-transduced Jurkat as target cells. *Error bars* represent the SEM. Statistical significance was determined by using the Students *t* test (ns, p > 0.05; *p < 0.05; **p < 0.01)

The requirement of UNG2 and RPA32 for virus replication was then analyzed in Jurkat lymphoid T cells (Fig. 3). Viruses were produced in UNG2- or RPA32-depleted 293T cells and used for infection of Jurkat cells also depleted of either UNG2 or RPA32 (Fig. 3a). Of note, no apparent impact on cell proliferation and viability was observed in shUNG2- and shRPA32-tranduced cells (data not shown). As shown in Fig. 3b, d, the depletion of UNG2 in virus producing cells and target Jurkat T cells resulted in a net decrease of virus replication (red curve and red bars). Interestingly, both viruses produced in shLuc- or in shUNG2-transduced cells failed to replicate in UNG2-depleted Jurkat recipient cells (red dashed line and red slanted oblique bars), indicating that the absence of UNG2 expression is critical during several rounds of virus replication and dissemination in the target Jurkat T cells. In agreement, depletion of UNG2 in virus-producing cells only was not sufficient to impact virus replication in Jurkat control cells (black dashed line and black slanted oblique bars). Similarly, viruses produced in RPA32-depleted cells or shLuc-transduced cells failed to replicate efficiently in RPA32-depleted Jurkat target cells (Fig. 3c, e). Like in HeLa-CD4 cells, a significant decrease in virus infectivity measured in a single-round infection assay in target Jurkat cells was observed only when viruses were produced in UNG2- and RPA32-depleted cells (Fig. 3f), confirming that incorporation of UNG2 and RPA32 into viral particles is required for maintaining full HIV-1 infectivity in a single-round assay (7). In contrast, depletion of UNG2 and RPA32 in the target Jurkat cells had no effect on virus infectivity in this assay (Fig. 3f), confirming that the absence of UNG2 and RPA32 is only critical during several rounds of virus replication. These results confirm the positive impact of UNG2 and RPA32 for efficient HIV-1 replication in human cell-lines.

### UNG2 and RPA32 are expressed at low level in HIV-1 primary target cells

**Fig. 4:**
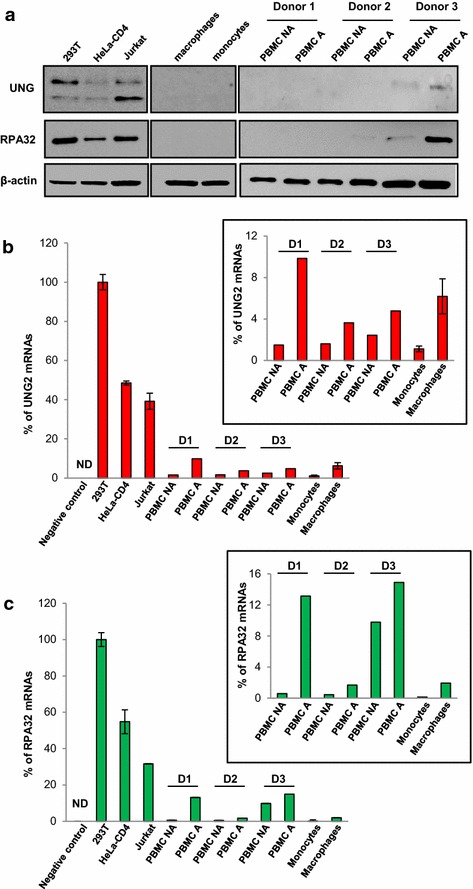
UNG2 and RPA32 expression in human cell lines and primary cells. Primary monocytes and PBMCs were isolated from blood of healthy donors. While PBMCs were activated in culture medium supplemented with PHA for 72 h and then IL-2 for 48 h, monocytes were differentiated in macrophages for 7 days in culture medium supplemented with M-CSF. **a** UNG2 and RPA32 protein expression. Equivalent amounts of proteins from total lysates of 293T, HeLa-CD4 and Jurkat cells, monocytes and macrophages, and non-activated and activated PBMCs (NA and A, respectively) were resolved by SDS-PAGE and analyzed by Western blotting with anti-UNG, anti-RPA32 and anti-β-actin antibodies. **b**, **c** Quantification of UNG2 and RPA32 mRNA expression. RNA was extracted and purified from 293T, HeLa-CD4, Jurkat cells, monocytes, macrophages, and non-activated and activated PBMCs, and UNG2 (**b**) and RPA32 (**c**) mRNA expression levels were then measured by quantitative RT-qPCR. Results are expressed as the percentage of UNG2 and RPA32 mRNA copies relative to those measured from 293T cell RNA extract. Data shown are means of three independent experiments (293T, HeLa and Jurkat cells) or from three independent donors for PBMCs and MDMs, performed in duplicate. *Error bars* represent 1 standard deviation (SD) from the mean. The *inset graphs* focus on mRNA expression levels in the primary cells. Negative control corresponds to 293T RNA extract processed without reverse transcriptase in the reaction mixture. *ND* no detection

Because most of the studies regarding the potential role of UNG2 for HIV-1 infectivity and replication have been performed using established human cell-lines [7, 10, 12, 29], we then focused our study on the impact of endogenous UNG2 as well as RPA32 in the context of primary target cells of HIV-1, including PBMCs and macrophages isolated from blood of healthy donors. First, we analyzed UNG2 and RPA32 expression in human cell lines, primary monocytes, monocyte-derived macrophages (MDMs), as well as activated and non-activated PBMCs both at the protein and mRNA transcript levels. As evidenced by Western blot analysis (Fig. 4a), all the primary cells studied expressed very low or undetectable levels of UNG2 compared to different cell lines such as 293T, HeLa-CD4 cells and the Jurkat CD4 T-lymphoid cell-line. In PBMCs (middle panels), different donors were analyzed and a variability in protein expression was observed. We failed to detect a UNG2 band in cells from two different donors (donors 1 and 2) whereas a band corresponding to the 39 kDa UNG2 protein was observed from one donor (donor 3). Similarly, quantification of UNG2 mRNA evaluated by semi-quantitative qRT-PCR on the same primary cells and cell-lines confirmed the low level of UNG2 transcripts in HIV-1 primary target cells compared to established human cell-lines (Fig. 4b). However, low but significant expression of UNG2 transcripts was detected, especially in activated PBMCs from the three donors and macrophages compared to the negative controls (Fig. 4b, inset). Regarding RPA32 expression, the protein was also undetectable in myeloid lineages while it was highly expressed in cell lines (Fig. 4a). In PBMCs depleted of the myeloid cells but containing the lymphoid cells, RPA32 was again detected from the cells of donor 3, where the protein is well expressed. In cells of donor 2, RPA32 protein was only weakly detected in cells activated with PHA and IL-2. For both donors 2 and 3, we noticed that activated PBMCs displayed more RPA32 protein expression than non-activated cells. qRT-PCR analysis of RPA32 mRNA content in cell lines as well as in primary cells confirmed that the RPA32 gene is less weakly transcribed in PBMCs, especially when cells were activated, compared to myeloid cells (Fig. 4c). However, the RPA32 transcript level remains very low compared to the 293T, HeLa and Jurkat cell lines. These results confirm that UNG2 and RPA32, two cellular proteins involved in DNA repair and replication, are expressed at lower levels in primary lymphoid and myeloid cells than in established cell lines.

### Impact of UNG2 and RPA32 on HIV-1 replication in PBMCs

**Fig. 5:**
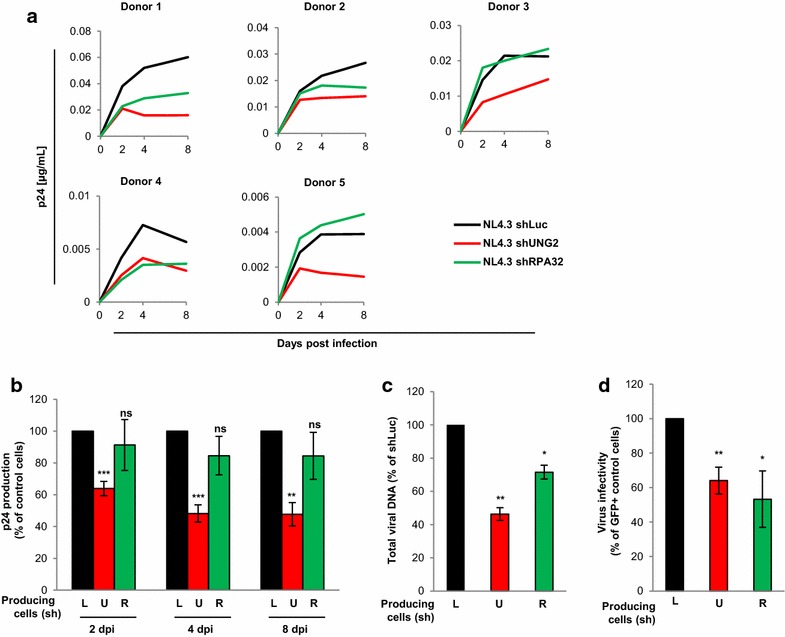
Impact of UNG2 and RPA32 on HIV-1 replication in PBMCs. **a**–**c** Viral replication. Replication-competent viruses were produced in shLuc- (*black curves* and *bars*), shUNG2- (*red curves* and *bars*) or shRPA32- (*green curves* and *bars*) transduced 293T cells, normalized for p24, and then used for infection in duplicate of PBMCs from five different healthy blood donors. Aliquots of PBMC culture supernatant were collected 2, 4 and 8 days after infection for p24 quantification. In **a**, the individual kinetics of replication in PBMCs from the five healthy donors are shown. In **b**, results are expressed as the percentage of p24 production at each time point relative to that of PBMCs infected with viruses produced in shLuc-transduced (*black bars*) 293T cells. Values are the means of two independent experiments performed on PBMCs from the five healthy donors. In **c**, PBMCs were collected 7 h after infection, subjected to DNA purification, and total viral DNA was quantified via qPCR using specific primers for *U5*-*gag*. Results are expressed as the percentage of total viral DNA relative to that of PBMCs infected with viruses produced in shLuc-transduced (*black bar*) cells. **d** Virus infectivity. GFP reporter viruses were produced in shUNG2-, shRPA32- or shLuc-transduced 293T cells as indicated, normalized for p24, and then used to infect PBMCs from three different donors. The percentage of GFP-positive infected cells was then measured by flow cytometry 60 h later. Viral infectivity was normalized to that of viruses produced in shLuc-transduced 293T cells. *Error bars* represent the SEM. Statistical significance was determined by using the Students *t* test (ns, p > 0.05; *p < 0.05; **p < 0.01; ***p < 0.001)

According to these observations, it seemed important to reevaluate the contribution of both UNG2 and RPA32 proteins on virus replication in the primary target cells of HIV-1. First, the replication of viruses produced in UNG2- and RPA32-depleted cells was then analyzed in PBMCs. X4-tropic viruses (NL4.3 strain) were produced in UNG2- or RPA32-depleted cells as well as in control cells and used to infect PBMCs isolated from different blood donors. As shown in Fig. 5a (red curves), depletion of UNG2 in virus-producing cells negatively impacted virus replication in PBMCs from the five donors. Summary and statistical analysis of the replication data measured for the different donors are shown in Fig. 5b. We observed a significant decrease of p24 production (about 50 %) when PBMCs were infected with viruses produced in UNG2-depleted cells. This defect in virus replication and production was evidenced as early as 2 days after infection and remained significant after 8 days (Fig. 5b, red bars), suggesting that the low level of UNG2 and RPA32 is not sufficient to restore long term efficient virus replication in PBMCs. In parallel, we analyzed the impact of RPA32 depletion during virus replication in PBMCs from the same donors. As shown in Fig. 5a (green curves), viruses produced in RPA32-depleted cells exhibited differential replication abilities depending on the cell donor. Compared to control viruses (black curves), a relative defect in virus replication was observed in PBMCs from three out of five donors (donors 1, 2 and 4), while replication in PBMCs from donors 3 and 5 was similar to that of the control viruses. Although a slight decrease of p24 production was continuously observed from viruses produced in RPA32-depleted cells during the 8 days of monitoring (Fig. 5b, green bars), this defect in virus replication was not statistically significant compared to the control viruses (black bars). These data regarding the role of RPA32 for HIV-1 replication could be linked to the differential level of protein expression measured in PBMCs from individual donors (see Fig. 4).

Since we previously showed that the defect of virus replication in UNG2-depleted human CD4-positive cell lines ([7], Fig. 2e) was related to a defect in the RT process, we quantified total viral DNA reverse transcripts 7 h after infection of PBMCs with viruses produced in UNG2- or RPA32-depleted cells. As shown in Fig. 5c, a significant reduction of the total viral DNA was observed with viruses produced in UNG2-depleted cells (red bar) compared to viruses produced in control cells (black bar). Interestingly, a slight but significant decrease in viral DNA synthesis was also revealed when PBMCs were infected with viruses produced in RPA32-depleted cells (Fig. 5c, green bar). Together, these results confirm that UNG2 and RPA32 depletion negatively impacted virus replication in PBMCs by influencing the efficiency of the RT process. Again, production in UNG2- or RPA32-depleted cells had a negative impact on virus infectivity measured in a single-round infection assay using PBMCs as target cells (Fig. 5d) confirming the requirement for UNG2 and RPA32 for optimal virus infectivity.

### UNG2 and RPA32 enhances HIV-1 replication in macrophages

**Fig. 6:**
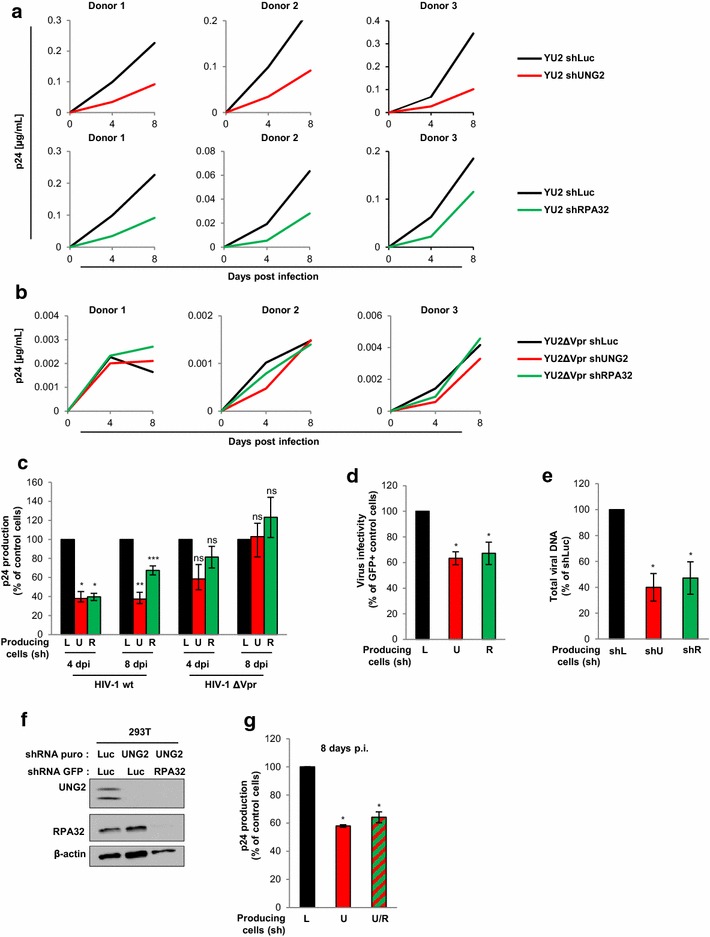
Impact of UNG2 and RPA32 on HIV-1 replication in macrophages. **a**–**c** Wild-type (**a**) or Δ*vpr* (**b**) replication-competent viruses were produced in shLuc- (*black curves* and *bars*), shUNG2- (*red curves* and *bars*) or shRPA32- (*green curves* and *bars*) transduced 293T cells, normalized for p24, and then used for infection in duplicate of MDMs from 3 different healthy donors. In **a** and **b**, aliquots of MDM cell culture supernatants were collected 4 and 8 days after infection for p24 quantification. The individual kinetics of replication in PBMCs from the three healthy donors are shown. In **c**, results are expressed as the percentage of p24 production at each time point relative to that of MDMs infected with wt or Δ*vpr* viruses produced in shLuc-transduced (*black bars*) cells. Values are the means of two independent experiments performed on MDMs from the two donors. **d** Virus infectivity in MDMs. Wild-type GFP reporter viruses were produced in shUNG2-, shRPA32- or shLuc-transduced 293T cells, normalized for p24, and then used to infect MDMs from three different donors. The percentages of GFP-positive infected cells were then measured by flow cytometry 60 h later. Viral infectivity was normalized to that of viruses produced in shLuc-transduced (*black bars*) 293T cells. **e** Replication-competent viruses were produced in shLuc-, shUNG2- or shRRA32-transduced 293T cells, normalized for p24, and then used for infection of MDMs from three different donors. MDM samples were collected 72 h after infection, subjected to DNA purification, and total viral DNA was quantified via qPCR using specific primers for *U5*-*gag*. Results are expressed as the percentage of total viral DNA relative to that of MDMs infected with viruses produced in shLuc-transduced (*black bar*) cells. **f** Double-depletion of UNG2 and RPA32 expression in virus-producing 293T cells. Cells were transduced with lentiviral vectors expressing shRNA against UNG2 or Luciferase and containing the gene for puromycin resistance, and with lentiviral vectors expressing shRNA against RPA32 or Luciferase and the GFP reporter gene. Lysates from shRNA-transduced cells were analyzed by Western blot using anti-UNG2, anti-RPA32 and anti-β-actin antibodies. **g** Replication-competent viruses were produced in shLuc/shLuc-GFP (*black bar*), in shUNG2/shLuc-GFP (*red bar*) or in shUNG2/shRPA32-GFP (*red* and *green hatched bar*) 293T cells, normalized for p24, and then used for infection of MDMs from three different healthy donors. The concentration of p24 after 8 days of infection was expressed as the percentage of p24 production relative to that of MDMs infected with viruses produced in shLuc-transduced (*black bar*) cells. *Error bars* represent the SEM. Statistical significance was determined using Students *t* test (ns, p > 0.05; *p < 0.05; **p < 0.01; ***p < 0.001)

Besides the CD4-positive T-cell population, macrophages are thought to be important in the persistence and pathogenesis of HIV-1 infection due to their widespread presence in various tissues and their contribution to long-lived reservoirs of virus-infected cells [2, 3]. We therefore investigated the replication of viruses produced in UNG2- or RPA32-depleted cells in macrophages. These cells also express low levels of UNG2 and RPA32 (see Fig. 4). R5-tropic viruses (YU-2 strain) were produced in UNG2- or RPA32-depleted cells and used to infect MDMs from different healthy donors. As shown in Fig. 6a, UNG2- (red curves) but also RPA32-depleted (green curves) virions failed to replicate efficiently in MDMs isolated from three blood donors. Compared to the p24 production monitored 4 and 8 days after infection of MDMs with control wild-type viruses (Fig. 6c, black bars), the p24 production from MDMs infected with UNG2- or RPA32-depleted viruses was significantly reduced (50–70 %) (red and green bars, respectively). Previously, we and others clearly showed that UNG2 is incorporated into virions through direct interaction with the viral Vpr protein (6–12), we similarly analyzed replication in MDMs of *vpr*-deleted viruses (ΔVpr) produced from UNG2- and RPA32-depleted cells (Fig. 6b, c). In contrast to wild-type viruses, no significant difference in viral replication was detected between ΔVpr viruses produced in control cells and UNG2- and RPA32-depleted ΔVpr viruses. These results indicate that the recruitment of UNG2 and RPA32 into virions favors virus dissemination in differentiated macrophages which express low levels of these two cellular proteins. Moreover, these cells could not counteract the negative effect of UNG2 depletion in the initial 293T cells. In agreement, infectivity of single-round viruses produced in UNG2- or RPA32-depleted cells was also impaired using MDMs as target cells (Fig. 6d).

As previously, we further investigated whether the impairment of replication in MDMs of UNG2- or -RPA32-depleted viruses was also linked to a RT defect during the establishment of infection. The total viral DNA reverse transcripts were measured 72 h after infection of MDMs with viruses produced from UNG2- or RPA32-depleted cells, and revealed a reduction of about 50–60 % of the total viral DNA synthesis compared to MDMs infected with control viruses (Fig. 6e). As observed in PBMCs, the absence of either UNG2 or RPA32 expression in virus-producing cells similarly decreases the efficiency of the RT process during viral replication in MDMs.

Finally, replication of viruses produced from cells depleted of both endogenous UNG2 and RPA32 proteins was evaluated in MDMs. 293T cells were thus co-transduced with two lentiviral vectors expressing specific shRNAs targeting UNG2 and RPA32, leading to efficient depletion of both proteins as evidenced by Western blot analysis (Fig. 6f). Replication-competent virus particles were produced in these double-depleted cells and used to challenge MDMs as previously. As shown in Fig. 6g, the replication impairment of viruses produced in cells depleted of both proteins (slanted red/green bar) was similar to the replication defect measured in MDMs infected with viruses produced in cells depleted of UNG2 only (red bar). The p24 concentration in MDM supernatant was indeed reduced of about 40–50 % for both viruses produced in shUNG2- or in shUNG2/shRPA32-tranduced cells 8 days after infection, showing that simultaneous depletion of UNG2 and RPA32 had no additional or synergistic effects compared to viruses produced in UNG2-depleted cells.

### UNG2 impacts on HIV-1 dissemination between T cells and macrophages

Because macrophages express low levels of both UNG2 and RPA32 proteins, we developed experimental systems to analyze how UNG2 and RPA32 expression could influence cell-free virus dissemination from T lymphocytes to macrophages or, reciprocally, from macrophages to T lymphocytes.

**Fig. 7:**
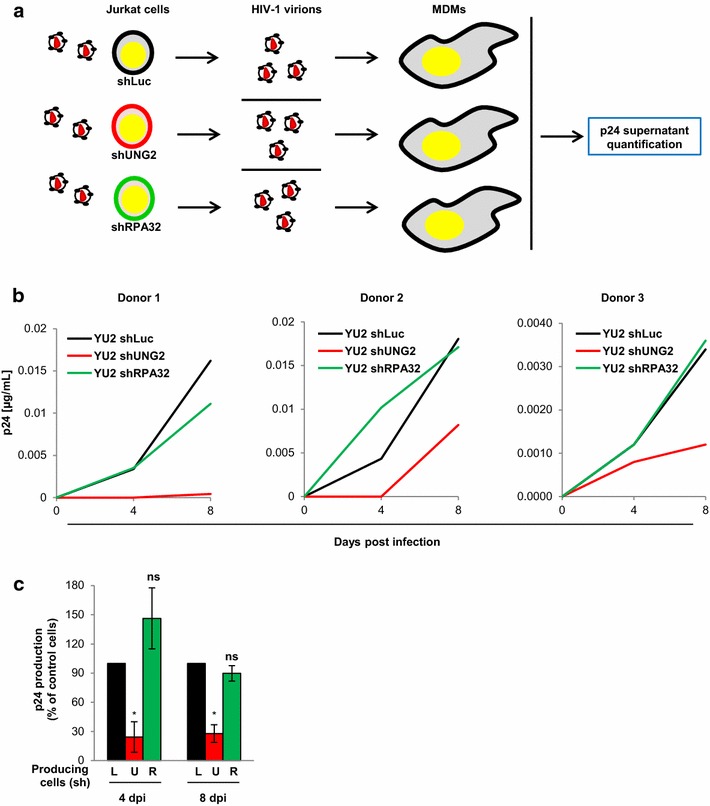
Impact of UNG2 and RPA32 for dissemination of cell-free virus particles between T cells and MDMs. **a** Schematic representation of the experimental system. shLuc, shUNG2- or shRPA32-transduced Jurkat cells were infected with HIV-1 (YU2 strain) expressing the VSV-G envelope, and the cell culture supernatant was then collected 3 days later. After p24 normalization, cell-free viruses produced by shRNA-transduced Jurkat cells were used for infection of MDMs, and virus production was monitored after infection. **b** and **c** Replication in MDMs of Jurkat cells-produced viruses. Replication-competent viruses were produced in shLuc-, shUNG2- or shRPA32-transduced Jurkat cells and then used to infect MDMs. Aliquots of cell culture supernatants were collected 4 and 8 days after infection for p24 quantification. In **b**, the individual kinetics of replication in MDMs from three different donors of viruses produced in shUNG2- or shRPA32-tranduced Jurkat cells are shown. In **c**, results are expressed as the percentage of p24 production at each time point relative to that of MDM cells infected with control viruses. Values are the means of two independent experiments performed with MDMs from three different donors. *Error bars* represent the SEM. Statistical significance was determined using Students *t* test (ns, p > 0.05; *p < 0.05)

First, we analyzed virus dissemination from T cells to MDMs as schematized in Fig. 7a, using UNG2- and RPA32-depleted Jurkat T cells as virus producing cells and MDMs expressing low or undetectable levels of UNG2 protein as target cells. Briefly, Jurkat cells were first transduced with shRNA targeting UNG2 or RPA32 and then infected with replication-competent HIV-1 co-expressing the vesicular stomatitis virus G (VSV-G) envelope to standardize the level of infected Jurkat cells. Three days later, virus particles were collected from the cell culture supernatant from shUNG2- or shRPA32-transduced Jurkat cells, normalized for viral p24, and used to infect MDMs. Virus replication in the target MDMs was measured 4 and 8 days later by monitoring p24 content in the cell culture supernatants. Three independent experiments using recipient MDMs from three different donors were performed in duplicate (Fig. 7b), and the replication data measured for the three donors is summarized in Fig. 7c. As expected, UNG2 depletion in virus-producing Jurkat T cells impaired virus dissemination in MDMs (red curves and bars). This impairment in virus replication was equivalent to the defect we previously observed in MDMs infected with viruses produced in UNG2-depleted 293T cells (see Fig. 6a, c). Surprisingly, depletion of RPA32 in virus-producing Jurkat T cells had no significant effect on virus replication in MDM recipient cells from the same donors (Fig. 7b, c, green curves and bars), suggesting that RPA32 is not required for virus dissemination specifically from Jurkat T cells toward macrophages.

**Fig. 8:**
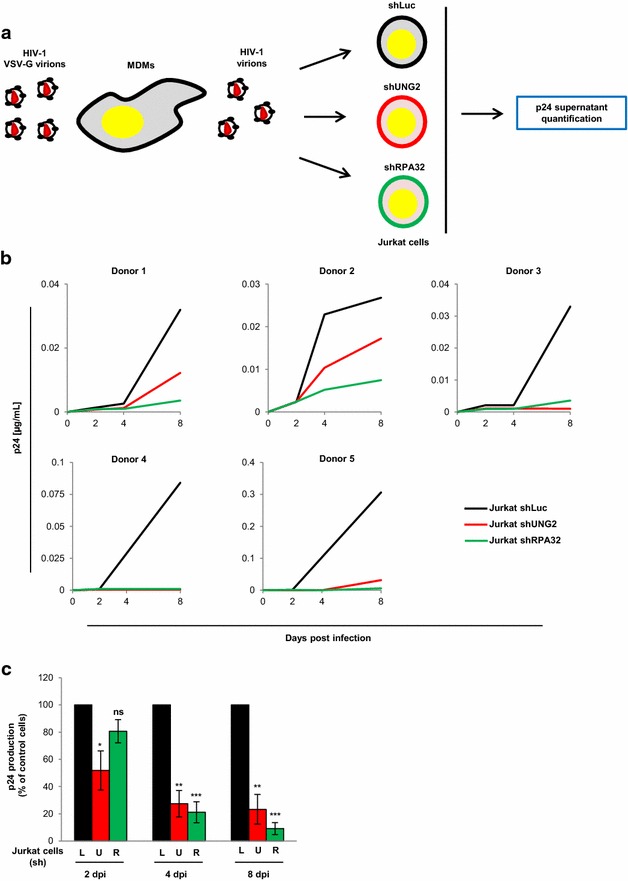
Impact of UNG2 and RPA32 for dissemination of cell-free virus particles between MDMs and Jurkat T cells. **a** Schematic representation of the experimental system. MDMs were infected with replication-competent HIV-1 (NL4.3 strain) co-expressing the VSV-G envelope, and the cell culture supernatant was then collected 8 days later. After p24 normalization, cell-free viruses produced by MDMs were used for infection of shLuc-, shUNG2- or shRPA32-transduced Jurkat cells, and virus production was monitored after infection. **b** and **c** Replication in Jurkat cells of MDMs-produced viruses. Replication-competent viruses were produced in MDMs and then used for infection of shLuc- (*black lines* and *bars*), shUNG2- (*red lines* and *bars*) or shRPA32- (*green lines* and *bars*) transduced Jurkat cells. Aliquots of cell culture supernatants were collected 2, 4 and 8 days after infection for p24 quantification. In **b**, the individual kinetics of replication in shRNA-tranduced Jurkat cells of viruses produced in MDMs from five different donors are shown. In **c**, results are expressed as the percentage of p24 production at each time point relative to that of shLuc-transduced Jurkat cells. Values are the means of two independent experiments performed with virus produced in MDMs from five different donors. *Error bars* represent the SEM. Statistical significance was determined using Students *t* test (ns, p > 0.05; *p < 0.05; **p < 0.01; ***p < 0.001)

Finally, dissemination of cell-free virus particles from macrophages toward T cells was analyzed using infected MDMs as virus-producing cells and UNG2- or RPA32-depleted Jurkat cells as target cells (schematized in Fig. 8a). MDMs were infected with replication-competent HIV-1 co-expressing the VSV-G envelope to increase and standardize the level of infected MDMs. After 8 days of infection, virus particles produced were collected from the MDM cell culture supernatant, normalized for p24 and used to infect Jurkat T cells previously transduced with shRNA targeting UNG2 or RPA32, as well as shLuc-transduced Jurkat cells as control cells. Viral replication was finally assessed in these recipient Jurkat cells by p24 monitoring in the cell culture supernatants at different days after infection. Independent experiments using viruses produced from MDMs isolated from five different donors were performed in duplicate (Fig. 8b), and the replication data measured for all the donors are summarized in Fig. 8c. Clearly, virus particles produced from macrophages replicated in control Jurkat cells, whereas replication of the same viruses in UNG2- or RPA32-depleted cells was severely affected (Fig. 8b). As summarized in Fig. 8c, virus production from shUNG2- or shRPA32-transduced Jurkat target cells led to an initial slight decrease of virus production measured 2 days after infection, but a 70–90 % decrease from UNG2- and RPA32-depleted T cells was measured 4 and 8 days after infection. These data confirm the positive impact of UNG2 and RPA32 for the long term replication of HIV-1 in target T cells.

Taken together, these results indicate that UNG2 is required for optimal replication and dissemination of cell-free virus particles between the target cells of HIV-1, while RPA32 is only required for dissemination from MDMs to target T cells.

## Discussion

We previously documented that incorporation of the UNG2 DNA repair protein into virus particles was required for modulation of the reverse transcription process [6, 30] resulting in a positive effect on HIV-1 infectivity and replication in established cell-lines [7]. However, this positive impact of UNG2 on reverse transcription and virus replication was independent of the enzymatic activity of the UNG2 protein. Here, we report results showing that the HIV-1 Vpr protein can form a complex with UNG2 and the RPA32 subunit of the RPA complex in virus-producing cells, and participate in the modulation of the reverse transcription process for optimal virus replication and dissemination between the different target cells of HIV-1.

While several previous studies showed that UNG2 was efficiently recruited into HIV-1 virions [8–10, 12] indicating that this recruitment had a positive influence on viral replication [8, 9], the role of UNG2 incorporation was challenged by other studies, suggesting a dispensable [10, 29] or detrimental [11, 12, 31] effect of UNG2 on virus replication. Because all the previous studies regarding a detrimental or dispensable role of UNG2 in HIV-1 replication have been performed using established human cell-lines, we investigated and analyzed here the role of UNG2, and also RPA32, on virus replication and dissemination in HIV-1 primary target cells. In agreement with previous reports [8, 9, 32, 33], our data confirm the low levels of UNG2 and RPA32 expression in primary hematopoietic cells including PBMCs and MDMs, both at the protein and mRNA transcript levels, compared to established human cell-lines. Recent biochemical analyses performed in primary cells also confirmed that CD4-positive T cells and MDMs expressed modest and low levels of UNG activity, respectively [33]. While the UNG2 and RPA32 proteins were not detectable in MDMs, expression in PBMCs seemed more variable and certainly higher than in MDMs. However, low but significant levels of UNG2 and RPA32 mRNAs were detected both in PBMCs and MDMs.

We now highlighted that UNG2 and RPA32 facilitated efficient virus replication and dissemination in PBMCs and macrophages. In these primary cells, the low level of UNG2 and RPA32 proteins did not allow the viruses to restore an efficient potential of replication. It was previously shown by Priet et al. [8] and Jones et al. [9] that UNG2 was required for efficient infection of macrophages with R5 viruses, but our data indicate that both X4 and R5 viruses replicated more efficiently in PBMCs and macrophages, respectively, when UNG2, and also RPA32, were expressed in virus-producing cells. These more efficient virus replication and dissemination were linked to a specific role of both UNG2 and RPA32 on reverse transcription, since we show here that a significant defect in the total viral DNA synthesis was systematically evidenced in these primary target cells when viruses were produced from UNG2- and RPA32-depleted cells. In addition, similar impairment of virus replication in macrophages was observed for viruses produced in cells simultaneously depleted of both cellular proteins, suggesting that these two proteins act in the same pathway for the control of HIV-1 replication and dissemination.

Differentiated macrophages residing in different host tissues, including the CNS and lymphoid tissues, are long-lived cell targets for productive viral replication. In order to analyze how UNG2 and RPA32 may influence dissemination of cell-free HIV-1 particles between macrophages and T lymphocytes, we developed experimental systems to show that viruses produced in macrophages, expressing low levels of UNG2 and RPA32, failed to replicate efficiently in UNG2- or RPA32-depleted Jurkat T cells. Similarly, viruses produced in UNG2-depleted Jurkat T cells did not replicate efficiently in MDMs confirming the positive role of UNG2 for dissemination of X4- and R5-tropic strains [8, 9]. While we failed to obtain substantial and sustain depletion of UNG2 and RPA32 in either MDMs or PBMCs in order to analyze their role for virus dissemination between HIV-1 primary target cells, our results suggest that UNG2 and RPA32, depending of the activation status of PBMCs and MDMs, may modulate virus spreading from and toward macrophages through positive regulation of the reverse transcription process. These observations suggest that UNG2 and RPA32 can thus contribute to virus dissemination and establishment of persistent reservoirs of virus-infected MDMs in different host tissues during the natural course of HIV-1 infection ([1], for review).

The determinants of UNG2 required for direct interaction with RPA32 [16, 18, 19, 34] are located in the same N-terminal region that the determinants involved in the modulation of the viral DNA mutation frequency [7]. Since Vpr interacts directly with UNG2 through recognition of a WxxF motif found in the C-terminal part of the protein [6, 35], UNG2 might thus recruit Vpr and RPA32 simultaneously in a trimolecular complex (see Fig. 1d), as evidenced here in vitro using recombinant proteins but also with endogenous cellular proteins by co-immunoprecipitation assay. In addition, it would be interesting to analyze whether the two other subunits of RPA (i.e., RPA70 and RPA14) could also be recruited together with RPA32 in this complex.

The RPA complex plays key roles in different DNA repair pathways such as repair of DNA double-strand breaks by homologous recombination [36, 37] during post-replicative base excision repair (BER) in association with UNG2 [34, 38]. As a ssDNA binding protein, RPA32 is directly involved in the control of the assembly of the DNA repair machinery when DNA damage pathway signaling is engaged [39, 40]. Both RPA32 and UNG2 are thus present in the same replication foci [34], where the RPA complex recruits and enhances the ability of UNG2 to remove uracil in ssDNA at the replication fork [16, 18, 19, 34]. A similar involvement of both UNG2 and RPA32 proteins for efficient removal of uracil residues during viral DNA synthesis in primary HIV-1 target cells could be postulated. However, we previously showed that this positive impact of UNG2 on reverse transcription was related to a non-canonical scaffolding mechanism independent of the catalytic activity of the enzyme [7]. Interestingly, several reports accumulated evidences for such a scaffolding nonenzymatic role of UNG2 and viral-related UNG proteins. While UNG2 is absolutely required for efficient CSR and controlled SHM processes in B lymphocytes, controversial results showing that the enzymatic removal activity of the protein is dispensable for these activities [23, 24, 26, 41, 42]. It was suggested that the N-terminal domain of UNG2 may recognize DNA double-strand breaks and acts as an accessory site to provide a structural support for a scaffold function of the protein [27, 43]. Such a scaffolding function of UNG2, independent of its enzymatic activity but related to the N-terminal part of the protein, was also highlighted by its role during the assembly of the human centromere protein A (CENP-A) to sites of DNA damage [44]. Interestingly, CSR and assembly of CENP-A are both inhibited by ectopic expression of the wild-type HIV-1 Vpr protein via direct interaction with UNG2 suggesting that UNG2 may be recruited or act through its WxxF motif during these processes [26, 44].

Furthermore, UNG proteins encoded by numerous viruses, such as *Poxviridae* and *Herpesviridae*, are required for efficient virus replication in their respective primary target cells through a mechanism independent of the uracil-excising activity of their UNG protein [45–48]. For example, it was recently showed that the BKRF3 UNG protein encoded by Epstein–Barr virus played a critical role in the viral DNA synthesis by recruiting cellular and viral proteins to replication sites, but this function was also independent of its enzymatic activity [49]. While we cannot formally exclude that UNG2 also acts on the HIV-1 reverse transcription process through an enzymatic-dependent mechanism, our results highlight a potential scaffolding role of UNG2 related to RPA32 binding. This nonenzymatic scaffolding function of cellular UNG2 would be important during HIV-1 dissemination. Nonetheless, further investigations should give access to the characterization of this novel role of UNG-related proteins in various biological activities such as CSR and SHM processes in B lymphocytes as well as DNA synthesis of several viruses, including HIV-1.

Our results strongly suggest that UNG2 acts as a scaffold protein recruiting RPA32 into virions and that this interaction is required during the UNG2-dependent mechanism of RT control. Interestingly, it was very well documented that the RPA trimeric complex directly participated and played a crucial role in DNA replication of the simian virus 40 (SV40) and other polyomaviruses ([22], for review). More recent reports showed that this protein complex was also involved, indirectly or directly through viral DNA binding, in DNA synthesis of numerous other viruses, including Eptein Barr and Herpes simplex herpesviridae, adenovirus as well as polyoma- and papillomaviruses [45–48, 50–52]. During SV40 replication, RPA must act at different steps including the opening of the double strand DNA and the stabilization of the single strand DNA [22]. Although the exact mechanism of the RPA complex during the HIV-1 DNA synthesis needs to be specifically determined, we can hypothesize that RPA allows both (1) for efficient recruitment and processivity of the viral RT, and (2) for protection of the single strand viral DNA from degradation by direct binding. Indeed, it was also reported that the RPA complex was able to inhibit the deamination activity and the processivity of the APOBEC3G cytidine deaminase, suggesting that RPA plays a role in DNA protection from the editing activity of APOBEC3 proteins [53]. This last observation could support a model in which the recruitment of RPA32 by Vpr and UNG2 at the site of RT leads to the binding of RPA32 to the negative viral DNA for protection from deamination and nuclease activities. However, this protective role of RPA32, particularly against APOBEC3G activity, has to be more explored and might explain why the presence of Vpr can limit the APOBEC3G effect as reported before [11]. In agreement with a role of the RPA32 subunit of the RPA trimeric complex in HIV-1 DNA synthesis and viral replication, it was also previously shown that the RPA complex had a positive impact, at least in vitro, on the efficiency of the HIV-1 reverse transcription [54, 55].

## Conclusions

The results reported here indicate that cellular UNG2 and RPA32 facilitate optimal virus replication and dissemination in PBMCs and macrophages through positive modulation of the reverse transcription process. Interestingly, and in favor of a positive impact of the endogenous UNG2 for virus replication, it has been recently shown that the DNA synthesis of the human hepatitis B virus (HBV) was also facilitated in a UNG2-dependent manner in primary hepatocytes [56, 57]. In this model, UNG2 may counteract APOBEC-induced hypermutation of the HBV genome. Similarly, our results argue for a positive role of UNG2 and RPA for optimal viral DNA synthesis and virus dissemination between the primary target cells of HIV-1. These cellular proteins may thus contribute to virus dissemination and the establishment of viral reservoirs in different host tissues during the natural course of HIV-1 infection.

## Methods

### Vectors and expression plasmids

Vectors for expression of the HA-tagged forms of the wild-type UNG2 and Vpr proteins have been described previously [7, 58]. For the plasmid encoding HA-tagged RPA32, the RPA32 coding sequence was amplified by PCR with specific primers from the pGAD-RPA32 plasmid described previously [19], and the PCR product was then subcloned into the BamHI and XhoI restriction sites of the pAS1B plasmid as described [6]. The plasmid for expression in bacteria of UNG2 fused to GST has been described previously [6], while the plasmid encoding for GST-RPA32 (pGEX4T1-GST-RPA32) has been kindly provided by Yuan Jingsong from the University of Texas (USA). The pLKO.1 lentiviral vectors containing the gene for puromycine resistance and harboring shRNA targeting UNG2, RPA32 or the firefly luciferase (Luc) control were purchased from Sigma and have been described [7]. The pLKO.1-shLuc-GFP, and the pLKO.1-shRPA32-GFP vectors were constructed by replacing the puromycin resistance gene by the GFP reporter gene between the BamHI and the KpnI restriction sites of the parental pLKO.1-shUNG2 and -shRPA32 vectors [7]. The infectious clones of the NL4.3 and YU2 HIV-1 isolates (pNL4.3 and pYU2), as well as the plasmid encoding the VSV-G envelope glycoprotein, have been described [6, 7, 30].

### Cell culture and transfection

Jurkat T cells were maintained in Roswell Park Memorial Institute Medium (RPMI) while 293T cells and HeLa cells stably expressing CD4 (HeLa-CD4 cells) were grown in Dulbecco Minimal Essential Medium supplemented with 10 % fetal calf serum (FCS), 100 IU penicillin/mL and 100 µg streptomycin/mL (Invitrogen); shRNA-transduced Jurkat and 293T cells were maintained in complete medium containing 1 µg/mL puromycin (Invitrogen). Human monocytes and PBMCs were isolated from blood of healthy volunteers (Etablissement Français du Sang, Hôpital Saint-Antoine, Paris, France) by density gradient sedimentation in Ficoll (GE Healthcare) followed by adhesion-selection for 2 h at 37 °C. After extensive washing, monocytes were differentiated in macrophages (MDMs) for 10 days in complete culture medium RPMI 1640 supplemented with 20 % FCS, 100 IU penicillin/mL, 100 µg streptomycin/mL (Invitrogen) and 10 ng/mL of macrophage colony-stimulating factor (M-CSF) (Miltenyi Biotec). For activation of PBMCs, cells were grown in complete RPMI medium supplemented with phytohemagglutinin (PHA) (5 µg/mL) for 72 h and then resuspended in complete medium containing 10 ng/mL of IL-2. All cells were grown at 37 °C under 5 % CO_2_. For virus production, immunoprecipitation and pulldown assays, 293T cells were transfected using calcium phosphate DNA precipitation technique as described [6, 7, 59]. For lentiviral vector production, 293T cells were transfected using Jet Pei reagent (Polyplus Transfection) according to the manufacturer’s instructions.

### Pulldown and immunoprecipitation assays, and immunoblot analysis

The pulldown assay for analyzing interactions between UNG2, RPA32 and Vpr was performed as previously described [6, 60]. Briefly, GST-UNG2, GST-RPA32 and GST were produced in *E. coli*, strain BL21, as described [60]. 5 µg of recombinant GST, GST-UNG2 or GST-RPA32 were immobilized on 15 µL of GSH-Sepharose beads (GE Healthcare). Beads were washed and then incubated with 500 µg of lysate from transfected 293T cells as described previously [60, 61]. Bound material was resolved by sodium dodecyl sulfate–polyacrylamide gel electrophoresis (SDS-PAGE) on 12 % acrylamide precast gels (BioRad) and then analyzed by Western blotting with anti-HA (3F10, Roche) and anti-β-actin (Sigma) antibodies.

For immunoprecipitation assay, 293T cells expressing HA-Vpr were lysed as described previously [30]. After quantification with Bradford (Bio-Rad), 500 µg of cell lysate proteins were incubated with 30 µL of anti-HA affinity matrice (clone 3F10, Roche) for 2 h under gentle shaking at 4 °C. Elution from beads was carried out by incubation in 30 µL of 1× Laemmli buffer containing DTT for 10 min at 95 °C. 20 µg of the protein lysates and 15 µL of the precipitate were then resolved by SDS-PAGE on 12 % acrylamide precast gels (BioRad). Immunoprecipitate and cell lysate proteins were then analyzed by Western blotting with anti-HA (3F10, Roche), anti-UNG2 (clone 2C12, Origene), anti-RPA32 (clone RPA3-19, Abcam) and anti-β-actin (Sigma) antibodies.

For analysis of endogenous UNG2 and RPA32 expression in the different cell-lines and primary cells, as well as in transduced cells, cells were lysed using a NP40 buffer containing a protease inhibitor (Roche) by incubation for 30 min on a wheel at 4 °C. After spinning, supernatant was collected and the concentration of proteins was quantified by Bradford analysis using the manufacturer’s protocol (BioRad). Cell lysates were then resolved by SDS-PAGE and analyzed by Western blotting using anti-UNG2 (clone 2C12, Origene), anti-RPA32 (clone RPA3-19, Abcam) and anti-β-actin (Sigma) antibodies.

### Quantification of UNG2 and RPA32 mRNA by qRT-PCR analysis

RNA of cell lines and primary cells was extracted using the Pure link RNA mini kit (Ambion) according to manufacturer’s instructions. The reverse transcription was performed on equal amount of mRNA using the Maxima reverse transcriptase (Thermo Scientific) according to the manufacturer’s instructions. The RT reaction was processed as follow: 10 min at 25 °C, 15 min at 50 °C and 5 min at 85 °C. Then, UNG2 and RPA32 cDNAs were quantified by the LightCycler 480 qPCR system (Roche Applied Science). For UNG2 cDNA amplification, we used as forward primer 5′-GCCAGAAGACGCTCTACTCC-3′ and as reverse primer 5′-GCATCTCCGCTTTCCTCA-3′ which are specific for UNG2 and do not amplify UNG1. For RPA32 cDNA amplification, we used as forward primer 5′-AGGCCACCTGAGATCTTTTC-3′ and as reverse primer 5′-GGCTTTGCTTAGTACCATGTG-3′. Each PCR reaction contains 1X SYBR Green I Master mix (Roche), 100 nM of each primer and 1 µL of the RT product. For absolute quantification, we used dilutions of genomic DNA which contains known copies of genome and used as an internal control. Results were expressed as the percentage of UNG2 and RPA32 mRNA copies relative to those measured from 293T cell RNA extract.

### UNG2-, RPA32- and UNG2/RPA32-depleted cells

VSV-G-pseudotyped lentiviral particles (LVPs) harboring shRNA targeting Luc (shLuc), UNG2 (shUNG2), or RPA32 (shRPA32) were produced in 293T cells as described previously [7]. LVPs were then used to transduce 293T, HeLa-CD4 or Jurkat cells, and the levels of UNG2 or RPA32 protein expression were assessed by Western blot as previously [7]. For double-depletion of UNG2 and RPA32, 293T cells were first transduced with LVPs harboring the shRNA targeting UNG2 and the puromycin resistance gene. After selection in cell culture medium containing puromycin as described previously [7], shUNG2-tranduced cells were transduced with LVPs harboring the shRNA targeting RPA32 and the GFP encoding sequence, and GFP-positive cells were sorted 72 h later using the BD FACSJAZZ cell sorter.

### Virus production, replication and infectivity assays

Replication-competent HIV-1 (NL4.3 or YU2 strains) were produced as previously described in shRNA-transduced 293T cells by transfection with pNL4.3 or pYU2 molecular clones [7], and the plasmid for expression VSV-G was added to the DNA mixture when indicated. For HIV-1 replication monitoring, HeLa-CD4 cells (2 × 10^5^), Jurkat cells (3 × 10^5^), MDMs (1 × 10^6^) or PHA/IL-2-activated PBMCs (2 × 10^6^) were seeded and infected in 6-well plates with 200 ng of viral p24. Cell culture supernatants were then collected 2, 4, and 8 days after infection (depending on the experiments) for p24 concentration measurement by enzyme-linked immunosorbent assays (ELISA) as described [7]. To monitor HIV-1 infectivity, single-round-infection viruses carrying the GFP gene were produced as previously described [7] in shRNA-transduced 293T cells followed by transfection with a DNA mix containing the HIV-1-packaging plasmid (pCMVDR8.2), the HIV-1 vector encoding GFP (pHIvec2.GFP), the plasmid encoding the HIV-1 envelope glycoproteins of the YU-2 isolate. The supernatant was then collected, filtered, and ultracentrifuged to pellet viruses as described previously [7]. For single-round infection experiments, HeLa-CD4 cells (2 × 10^5^), Jurkat cells (3 × 10^5^), MDMs (1 × 10^6^) or PHA/IL-2-activated PBMCs (2 × 10^6^) were seeded and infected in 6-well plates with 500 ng of p24. Cells were cultured at 37 °C for 60 h and samples were then fixed in 1 % paraformaldehyde (Sigma-Aldrich) and data were collected on a Cytomix FC500 cytometer (Beckman-Coulter). The percentage of GFP-positive cells was analyzed using the RXP analysis software. Viral infectivity was calculated by normalizing the percentage of GFP-positive cells to that obtained in cells infected with viruses produced in shLuc-transduced 293T cells.

### Quantification of total viral DNA reverse transcripts

One day prior to infection, HeLa-CD4 cells (2 × 10^5^), Jurkat cells (2 × 10^5^), MDMs (1 × 10^6^) or activated PBMCs (2 × 10^6^) were plated in 6-well plates. Before infection, replication-competent viruses were incubated with DNAseI (Roche) for 1 h at 37 °C, and 0.5 µg (or 1 µg for PBMCs) of p24 was then used for infection. 3 h after infection (or 24 h for MDMs), viruses were washed off and the cells were subsequently incubated at 37 °C in complete medium supplemented with 0.5 µM saquinavir in order to restrict viral replication to a single cycle. For HeLa-CD4, Jurkat cells and PBMCs, cell samples were collected 7 h after infection and DNA was extracted using the QIAamp DNA Blood Mini Kit (Qiagen) according to the manufacturer’s protocol. For MDMs, DNA extraction was carried out 72 h after infection as described [62]. The total level of HIV-1 DNA reverse-transcripts was quantified via the LightCycler 480 qPCR system (Roche Applied Science) as previously described [7, 63]. Briefly, the quantitative PCR for total HIV-1 DNA was carried out using primers targeting the *U5*-*gag* region within the HIV-1 genomic sequence in a 10-µL final volume consisting of 2X FastStartDNA Tag polymerase (Roche) and 0.3 µM of sense MH532 (5′-TGTGTGCCCGTCTGTTGTGT-3′) and antisense MH531 (5′-GAGTCCTGCGTCGAGAG ATC-3′) primers (TIB MolBiol). The fluorescent probe primers 5′-LC640-TCTCTAGCAGT GGCGCCCGAACAG-PH and 5′-CCCTCAGACCCTTTTAGTCAGTGTGGAA-FL were used at a concentration of 0.2 µM. Total DNA was expressed as copy numbers per cell, with the DNA template normalized by β-globin gene amplification using a LightCycler control kit DNA (Roche).
